# Alginate-Encapsulated Mycobacteriophage: A Potential Approach for the Management of Intestinal Mycobacterial Disease

**DOI:** 10.3390/v15122290

**Published:** 2023-11-22

**Authors:** Laura Michelle O’Connell, Aidan Coffey, Jim O’Mahony

**Affiliations:** Biological Sciences Department, Munster Technological University Bishopstown Campus, T12 P928 Cork, Ireland

**Keywords:** mycobacteriophage, sodium alginate, encapsulation, simulated gastrointestinal environments, phage therapy

## Abstract

Encapsulated medication is a common method of administering therapeutic treatments. As researchers explore alternative therapies, it is likely that encapsulation will remain a feature of these novel treatments, particularly when routes of delivery are considered. For instance, alginate-encapsulation is often favoured where gastric digestion poses an obstacle. When exposed to cations (namely Ca^2+^), alginate readily forms gels that are resilient to acidic conditions and readily dissociate in response to mid-range pH. This action can be extremely valuable for the encapsulation of phages. The efficient delivery of phages to the intestine is important when considering mycobacteriophage (MP) therapy (or MP prophylaxis) for disseminated mycobacterial infections and chronic gastroenteritis conditions. This study presents the design and in vitro validation of an alginate-encapsulated MP capable of releasing phages in a pH-dependent manner. Ultimately, it is shown that encapsulated phages pretreated with simulated gastric fluid (SGF) are capable of releasing viable phages into simulated intestinal fluid (SIF) and thereby reducing the mycobacterial numbers in spiked SIF by 90%. These findings suggest that alginate encapsulation may be a viable option for therapeutic and prophylactic approaches to the management of intestinal mycobacterial disease, such as Johne’s disease.

## 1. Introduction

Mycobacteriophage therapy (MPT) is likely to become an important alternative to antibiotic therapy for drug-resistant mycobacterial infections. Recent case studies have demonstrated MPT’s efficacy in clearing stubborn *Mycobacterium abscessus* infections in immunocompromised patients [1,2]. MPT may also prove useful as a replacement for prophylactic antibiotic regimens within the agriculture sector, as stricter policies regarding these regimens in food animals are enforced [3]. For example, farms with a history of *Mycobacterium avium* sbsp. *paratuberculosis* (MAP) infections—which results in Johne’s disease in ruminant animals [4]—may benefit from introducing prophylactic doses of MPs capable of preventing the spread and colonisation of MAP.

MAP takes advantage of the underdeveloped immune system of young ruminants [5]. Transmission is typically through the faecal-oral route and animals may be exposed to infection from their herd and through their environment [4]. Prophylactically treating at-risk livestock with MP could help prevent the development of chronic disease and potentially the frequency of passive shedding [6]. MAP has also long been considered one of many “triggering agents” involved in Crohn’s disease [7]. As research progresses, evidence continues to support that MAP infection or exposure is related to the prevalence of Crohn’s disease, with a regional prevalence of Crohn’s disease correlated to an environmental incidence of MAP [8].

There are inherent limitations to prophylactic and therapeutic treatments involving proteinaceous entities like phages. For instance, delivering phages orally results in exposure to the acidic conditions of the stomach before arriving in the intestines where MAP invades the epithelial barrier [5,9]. A logical approach to aiding the survival of phages in the stomach is encapsulation to create a barrier between the phages and the acidic environment. Alginate gels offer an ideal solution, as these gels are resilient to low pH and readily dissociate in neutral pHs [10]. This suggests that phages suspended in alginate matrices—and subsequently encapsulated in alginate gels—could be shielded from the acidic environment of the stomach, and later released into the intestinal environment as the pH gradually increases along the small intestine [11]. This was successfully demonstrated with various formulations of alginate-encapsulated phages (i.e., often containing additional gelling agents, e.g., chitosan [12]) in simulated gastrointestinal environments using phages targeting *Escherichia coli*, *Salmonella enterica*, and *Listeria monocytogenes* [13,14,15]. This study aims to demonstrate similar outcomes with MPs.

Specifically, this work aims to identify a robust bead configuration that allows a high level of phage retention during the encapsulation process, short term storage, and simulated gastric digestion, with rapid phage release in response to a simulated intestinal environment. These criteria are essential considerations for the basic functionality of the encapsulated phages. The data presented in this study show the stepwise strategies performed to achieve this goal using MP LOCARD and *Mycobacterium smegmatis* (a nonpathogenic mycobacterium used as a surrogate host in place of MAP). The encapsulation of a three-phage cocktail featuring LOCARD, staphylococcal phage K, and *E. coli* phage T4 is also demonstrated to indicate the potential to target several pathogens at once.

## 2. Materials and Methods

### 2.1. Reagents, Bacterial Strains, and Phages

All reagents used in this study were obtained from Sigma-Aldrich (Arklow, County Wicklow, Ireland) unless otherwise stated. *Mycobacterium smegmatis* mc2 155 (CP009494; the Leibniz Institute DSMZ, Braunschweig, Germany) was routinely cultured statically at 37 °C for 48 h in brain heart infusion broth (BHI). MP LOCARD (OQ383327; Munster Technological University’s phage collection, Cork, Ireland) was propagated by inoculating BHI broth with 100 μL of phage lysate and 500 μL of *M. smegmatis* mc2 155 and incubating this bacteria-phage suspension with agitation at 37 °C for 48 h. The resulting lysate was then filtered through a 0.45 μm syringe filter. For the purposes of creating a three-phage cocktail to demonstrate the creation of multivalent beads, *Staphylococcus aureus* DPC 5426 (DSM105272) and *E. coli* K-12 (NC000913.3) were cultured statically in BHI for 16–18 h at 37 °C. Staphylococcal phage K and *E. coli* phage T4 were propagated in BHI broth by adding 100 μL of phage lysate to 100 μL of bacterial culture and incubating the suspensions with agitation for 16–18 h at 37 °C. These phage propagations were also passed through a 0.45 μm filter to remove bacterial debris and cells. The bacterial and phage stocks were stored at −20 °C and −80 °C in 40% glycerol.

### 2.2. Encapsulation of LOCARD in Alginate

An alginate–LOCARD matrix was achieved by combining equal volumes of phage lysate adjusted to ~2 × 10^8^ PFU/mL with 1.5%, 2%, and 3% (*w*/*v*) sodium alginate to achieve final alginate concentrations of 0.75%, 1%, and 1.5%. The mixtures were added dropwise to 0.1 M CaCl_2_ using a syringe fitted with a 21-gauge needle to create small beads. The beads remained in the CaCl_2_ buffer for 15 min to permit gelation and then removed by passing the mixtures through sterilised conventional kitchen sieves. The homogeneity of the matrices and the titre of phages retained in the beads were examined by creating 1:10 dilutions—i.e., 1 mL of phage-alginate mixture or 1 g of beads was added to 9 mL “disruption buffer” (100 mM NaHCO_3_, 100 mM NaC_6_H_7_O_7_ 50 mM Tris-HCl, pH 7.5) [12]—and incubating them with vigorous agitation for 15 min at room temperature to dissociate the alginate beads. As the density of 0.5–3% alginate is approximately equal to that of water [16], it is presumed that 1 g of beads will contain a quantity of phages equivalent to 1 mL of the adjusted phage stock. The titres of the released phages from the dissociated alginate beads were determined via conventional plaque assay methods. The titres of the matrices were compared to the relevant beads to determine whether significant quantities of phages were lost during encapsulation. The matrix that appeared the most homogenous and featured the least phage loss was used as the “base matrix” for the modified matrices described below.

### 2.3. Introducing Sodium Bicarbonate and Chitosan to the Alginate Beads to Reinforce Encapsulation

**Figure 1:**
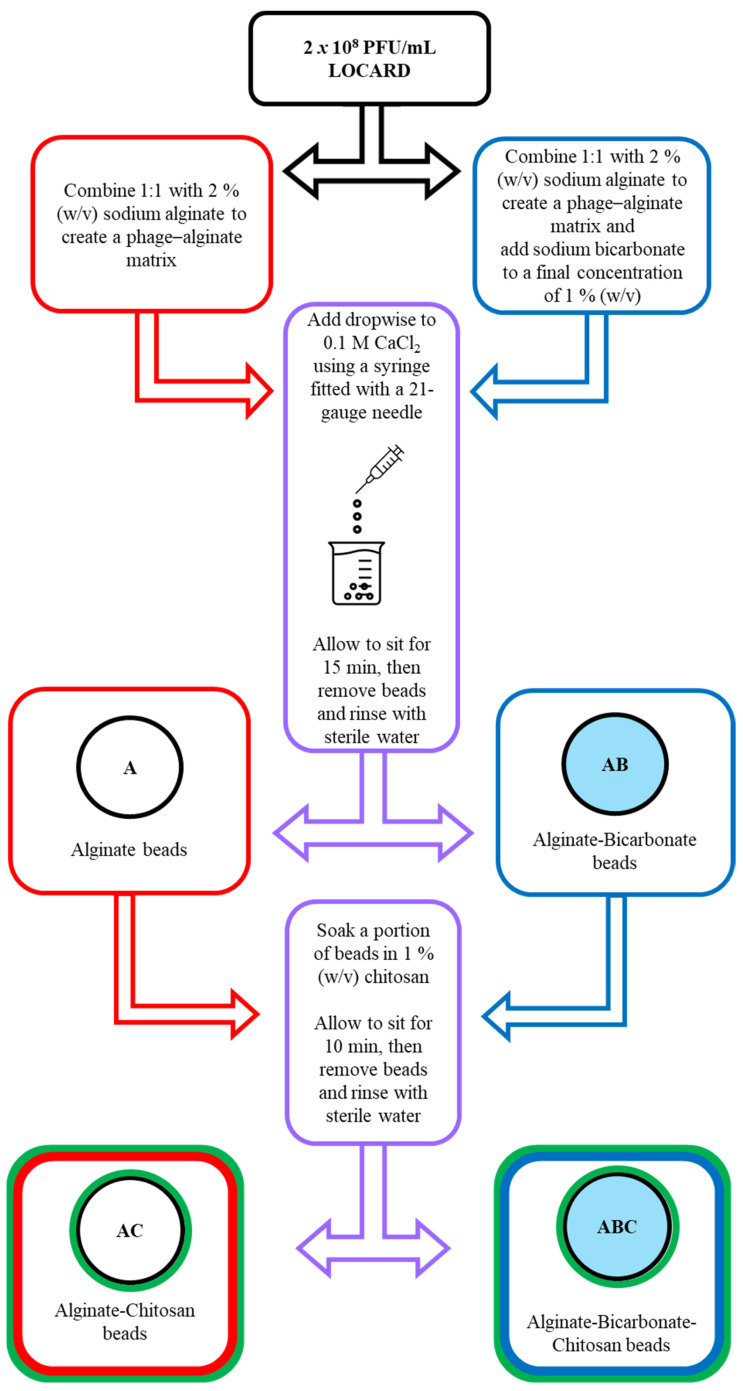
Flow chart describing the phage encapsulation process. LOCARD particles adjusted to 2 × 10^8^ PFU/mL are added to 2% sodium alginate. To a portion of this mixture, sodium bicarbonate is added to a final concentration of 1%. Both versions of the matrix are added dropwise to 0.1 M CaCl_2_ through a 21-gauge needle, resulting in alginate (A) and alginate-bicarbonate (AB) beads. A portion of A and AB beads are further treated with 1% chitosan to create alginate-chitosan (AC) and alginate-bicarbonate-chitosan (ABC) beads.

A solution of 1 × 10^8^ PFU/mL in 1% sodium alginate formed the base matrix required to create four varieties of alginate beads with the aim of identifying a particularly robust configuration (Figure 1). To half of this base mixture, sodium bicarbonate (powdered) was added to a final concentration of 1% (*w*/*v*) (i.e., 1:1 ratio of alginate: bicarbonate) [17], which should support the stability of the beads in acidic environments [18]. These mixtures were added dropwise to CaCl_2_, as described above, thus creating alginate (A) and alginate bicarbonate (AB) beads. The beads were rinsed with sterile water, and portions of the A and AB beads were submerged in 1% (*w*/*v*) chitosan (low viscosity, from shrimp shells), prepared in 1% (*v*/*v*) acetic acid for 10 min to add an additional layer to the bead structure [12], thereby creating alginate-chitosan (AC) and alginate-bicarbonate-chitosan beads (ABC). The physical appearance of the four bead types were compared by eye and photographically (12 MP sensor linked to a f/1.8-aperture lens, Samsung S20 FE, Three Ireland (Hutchinson) Ltd., Sir John Rogerson’s Quay, Dublin, Ireland). The sizes of the beads were determined using digital Vernier callipers (RS Pro Electronic Digital Callipers 150 mm Metric only, Radionics Ltd., Rialto, Dublin, Ireland). The beads were stored in 30 mL universal containers at 4 °C.

### 2.4. Examining the Quantity of Viable LOCARD Particles Retained in Each Variety of Beads

The quantity of viable phages retained in the beads was analysed to determine if the addition of bicarbonate and/or chitosan negatively affected phage retention. Phage retention was calculated by creating a 1:10 dilution of each bead type in disruption buffer and incubating them at room temperature with vigorous agitation, as described previously. The titres of released phages were compared to the phage stock (i.e., free phages) used to create the phage–alginate mixture.

### 2.5. Stability of the Encapsulated LOCARD during Short-Term Storage

A single batch of each bead type and a free phage control were stored in 100 mL sample cups for 28 days at 4 °C. On days 0, 14, and 28, triplicate samples were taken, and the encapsulated phages were released from the alginate matrices using disruption buffer. The titres recovered on each day were compared to the previous readings.

### 2.6. Release of LOCARD Particles in SIF

The release of phages in SIF (50 mM KH_2_PO_4_, 0.1% porcine bile extract, 0.4% pancreatin, pH 6.8) [13] was investigated by creating a 1:10 bead dilution, i.e., 1 g of beads was added to 9 mL SIF, and incubating these mixtures at 37 °C with gentle agitation at 60 rpm. Initially, phage release was monitored every 90 min for 6 h via plaque assay. Then, to pinpoint the moment of maximum phage release, the assay was repeated for a duration of 2 h, and every 15 min an aliquot was removed and assayed for the presence of viable phages. A positive control of free phages at a titre approximately equal to 1 g of beads was performed to determine whether the SIF negatively impacted LOCARD.

### 2.7. Exposure of Encapsulated LOCARD to SGF and Release of Phages from Gastrically Treated Beads in SIF

The survival of each version of the encapsulated LOCARD was compared against that of the free particles in SGF (0.2% NaCl, pH 2.5; ~1500 U/mL pepsin) [12]. Briefly, 1 mL of free phages or 1 g of beads was added to 9 mL of SGF and incubated at 37 °C with gentle agitation at 60 rpm. After 90 min, the effect of the SGF was terminated by removing the beads via sieving and rinsing them in sterile water. Disruption buffer was used to release the surviving phages. In the case of free phages, 1 mL of free phage-SGF suspension was immediately added to 9 mL of disruption buffer. The free phages and beads that were not exposed to the SGF were used as controls. The beads that retained the most LOCARD particles were selected for further experiments to determine if the beads treated with SGF behaved similarly to untreated beads in SIF. Essentially, the beads were incubated in SGF for 90 min, and then subjected to the 2 h phage release assay in SIF described in the previous section.

### 2.8. Reduction of Mycobacterial Numbers by Phages Released from Alginate Beads in SIF

The effect of the encapsulated phages on M. smegmatis in SIF was demonstrated by monitoring the viable mycobacterial population as the phages were released. The effect of the encapsulated phages that had been pre-exposed to SGF for 90 min was also examined. To spike the SIF, a culture of *M. smegmatis* was standardised to an OD_600nm_ of 0.08–0.1 (equates to approximately 1–1.5 × 10^8^ CFU/mL) and used to inoculate SIF containing beads (1 g in 9 mL) to achieve a multiplicity of infection (MOI) of ~1. The final mixture was incubated at 37 °C with gentle agitation at 60 rpm for 6 h. Every 90 min, a sample was taken, and the remaining population of *M. smegmatis* was determined using conventional spread plate and spot assay techniques [19]. A positive control was created by adding free phages to the spiked SIF, and a negative control of spiked SIF containing no phages (free or encapsulated) was used to verify that the bacteria were otherwise stable.

### 2.9. Encapsulation of a Phage Cocktail as a Preliminary Demonstration of Multivalent Phage Beads

To illustrate the possibility of encapsulating a cocktail of phages to maximise the effectiveness of therapy, a phage targeting a Gram-negative (*E. coli* phage T4) and a Gram-positive (staphylococcal phage K) host were combined with LOCARD prior to encapsulation. Each phage was adjusted to ~3 × 10^8^ PFU/mL and combined in equal volumes. This cocktail was then used to create beads containing ~5 × 10^7^ PFU/g of each phage. The titres of each phage in the cocktail and the corresponding beads were determined by completing plaque assays against each bacterial host. The phage retention was determined by comparing the titre of each phage in the cocktail to the titre of each phage released from the beads.

### 2.10. Statistical Analyses and Presentation of Data

Graphpad Prism (version 8.0.1 for Windows) was used to construct bar and line graphs from triplicate data. The mean of each triplicate was plotted with error bars representing the standard deviation. RStudio 2023.03.0 Build 386 was used to carry out statistical analyses. The Shapiro–Wilk test was used to determine the normality of the data. One-way ANOVAs were used to determine the presence of significance within the overall dataset, while Games–Howell (nonnormal data) or Dunnett (normal data) post hoc tests were used for pairwise comparisons to identify instances of significant differences between pairs. Unpaired *t*-tests were used to compare initial and recovered titres of phages from beads treated with simulated gastric fluid (SGF). Significance was illustrated on the graphs by asterisks as follows: * = *p* ≤ 0.05; ** = *p* ≤ 0.01; *** = *p* ≤ 0.001; **** = *p* ≤ 0.

## 3. Results

### 3.1. Encapsulation of LOCARD in an Alginate-Based Matrix

**Figure 2:**
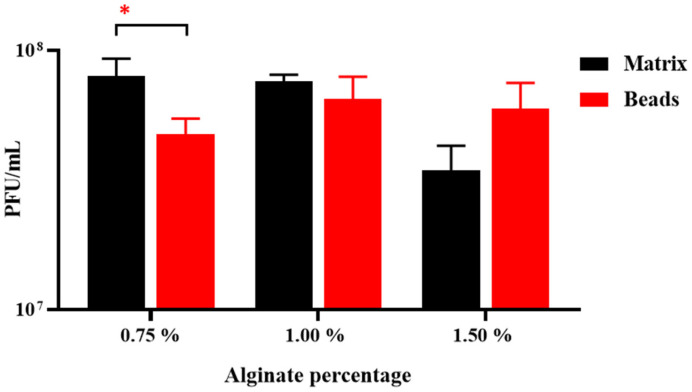
Homogeneity of the alginate matrices (0.5–1.5% *w*/*v* alginate) and quantity of phages retained in the corresponding beads. Significance is illustrated on the graphs by asterisks as follows: no asterisk = *p* ≥ 0.05; * = *p* ≤ 0.05.

The LOCARD particles were suspended in matrices containing 0.75%, 1%, and 1.5% alginate. To determine the homogeneity of the alginate matrices, the phage titre of three aliquots from each matrix was determined. The results indicated that a matrix featuring 1% alginate was the most homogeneous, as the standard deviation determined for the triplicate titres was the narrowest compared to that of the 0.75% and 1.5% matrices (Figure 2). The quantity of phages retained by the 1% alginate beads was not significantly different from the matrix, suggesting that a negligible number of phages was lost during the encapsulation process. On the other hand, significantly fewer phages were recovered from the 0.75% alginate beads, suggesting a statistically relevant number of phages was lost. While the difference in phage titres between the 1.5% alginate matrix and the 1.5% beads was not deemed statistically significant (*p* ≥ 0.05), it is apparent that a greater quantity of phages was recovered from the beads than the matrix (Figure 2). Considering the homogeneity of the matrix and the similar quantity of phages recovered from the beads, it was decided to proceed with 1% alginate as the base matrix for the modified beads analysed in the next section.

### 3.2. Introducing Sodium Bicarbonate and Chitosan to the Alginate Beads in an Effort to Reinforce Encapsulation

**Figure 3:**
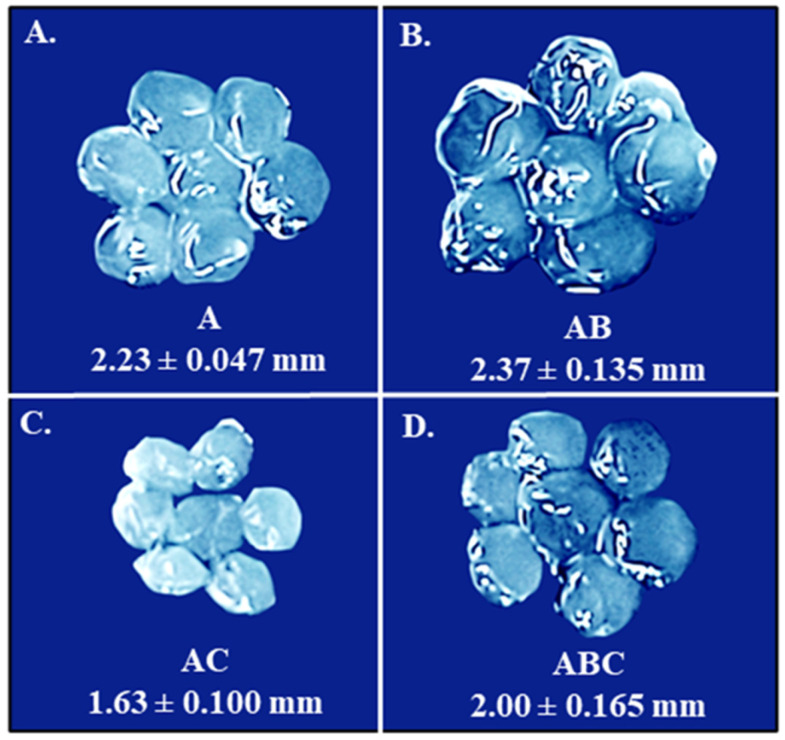
Images of the four varieties of beads. The images are presented proportionally, meaning that the differences in size shown in the figure are proportional to how they appear in situ. The average size of each kind of bead is indicated under the relevant image. (**A**). Alginate (A) beads. (**B**). Alginate-bicarbonate (AB) beads. (**C**). Alginate-chitosan (AC) beads. (**D**). Alginate-bicarbonate-chitosan (ABC) beads. Obvious physical and morphological differences are evident.

The base matrix was used to create four varieties of beads, the physical appearance of which is depicted in Figure 3. Hypothetically, the inclusion of bicarbonate and/or chitosan could reinforce the bead structure and make them more robust against acidic environments. The A beads are composed of only the base matrix (1 × 10^8^ PFU/mL LOCARD and 1% alginate), while the AB beads also include 1% bicarbonate. Treating these beads with 1% chitosan generated the AC and ABC beads, respectively. The A beads (Figure 3A) were more spherical than the AB beads (Figure 3B), which were slightly disc-shaped, gelatinous, and translucent, as though the physical properties of the calcium alginate matrix were altered by the bicarbonate. Treating A and AB beads with chitosan also seemed to alter their physical properties. The AC beads (Figure 3C) were smaller in size and became more matte and opaque. Interestingly, the chitosan solution made the ABC beads (Figure 3D) appear more similar to the A beads, although they were still more translucent.

### 3.3. Examining the Quantity of Viable LOCARD Particles Retained in Each Variety of Beads

**Figure 4:**
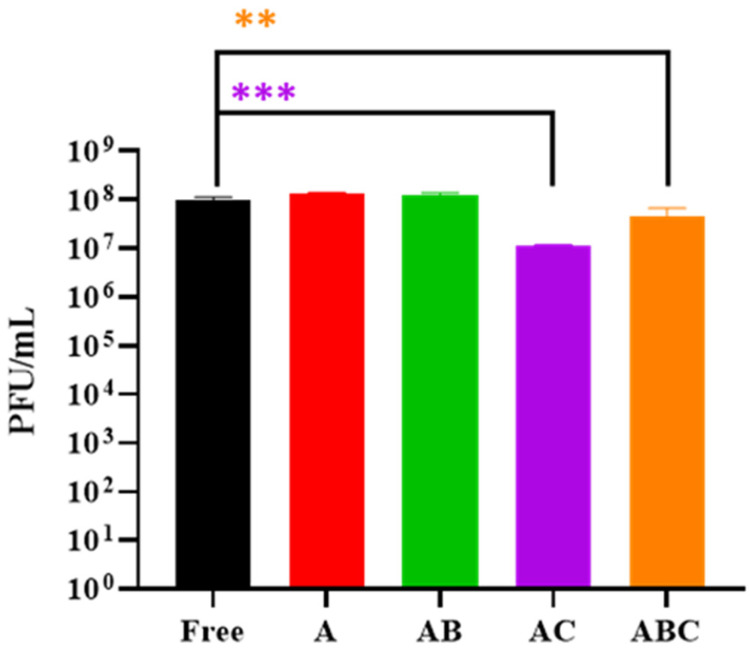
Phage titres recovered from beads treated with disruption buffer. “Free” refers to a control consisting of free phages that were used during the encapsulation process. A: Alginate beads. AB: Alginate-bicarbonate beads. AC: Alginate-chitosan beads. ABC: Alginate-bicarbonate-chitosan beads. Significance is illustrated on the graphs by asterisks as follows: no asterisk = *p* ≥ 0.05; ** = *p* ≤ 0.01; *** = *p* ≤ 0.001. The asterisk colour relates the significance value to the corresponding dataset.

The physical appearance of the four bead types at least partially related to how well each variant retained phages, as the chitosan treatment seems to be responsible for both a reduction in size and in phage titre (Figure 4). The PFU/g of the A and B beads was not deemed significantly different (*p* ≥ 0.05) to the PFU/mL of the free phage control, while the chitosan-treated beads contained significantly fewer phages, particularly the AC beads (*p* ≤ 0.001; Figure 4). The AC beads also retained significantly fewer phages than the A and AB beads (*p* ≤ 0.0 and *p* ≤ 0.001). This observation suggests the A and AB beads may be the best suited for therapy as they retain the most phages. However, the AC and ABC beads still contain a large number of phages, so no bead type was eliminated at this point and all four were analysed for the ability to release phages when exposed to a simulated intestinal environment.

### 3.4. Stability of the Encapsulated LOCARD during Short-Term Storage

**Figure 5:**
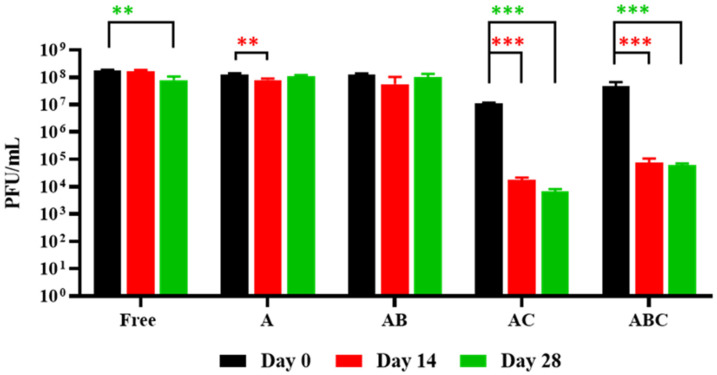
Phage retention during short-term storage. “Free” refers to a control consisting of free phages that were used during the encapsulation process. A: Alginate beads. AB: Alginate-bicarbonate (AB) beads. AC: Alginate-chitosan beads. ABC: Alginate-bicarbonate-chitosan beads. The composition of the beads affected retention as evidenced in the graph. Significance is illustrated on the graphs by asterisks as follows: no asterisk = *p* ≥ 0.05; ** = *p* ≤ 0.01; *** = *p* ≤ 0.001. The asterisk colour relates the significance value to the corresponding dataset.

To determine which bead variety retained the most phages during short-term storage, a single batch of each type of beads was stored for 28 days at 4 °C, alongside a free phage control. On day 0, 14, and 28, the phage titres remaining in the beads/control were enumerated and compared to the previous readings (Figure 5). Interestingly, the number of phages retained in the free phage control, A, and AB beads remained similar between Day 0 and Day 28, while the AC and ABC beads experienced a 3-log reduction (*p* ≤ 0.001) by Day 14. After Day 14, these beads did not experience any further phage loss. As the additional chitosan layer may prove beneficial during the subsequent assays (i.e., while not beneficial for storage, chitosan may be advantageous when the beads are exposed to low pH conditions), at this point it was determined once again not to eliminate any bead type. Instead, freshly prepared beads were consistently used.

### 3.5. Release of Phages from Each Bead Type When Exposed to SIF

**Figure 6:**
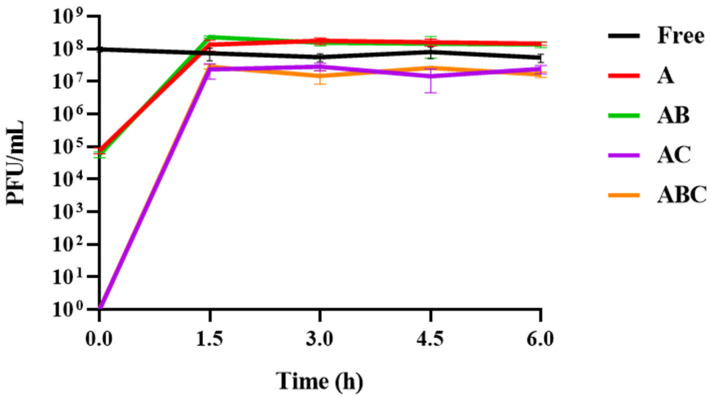
Phage release and stability in simulated intestinal fluid (SIF) over 6 h. “Free” refers to a control consisting of free phages that were used during the encapsulation process. On the *x*-axis, 0.0 refers to the moment the free phages or beads were added to the SIF, at which point a sample was immediately removed to determine the initial titres. A: Alginate beads. AB: Alginate-bicarbonate beads. AC: Alginate-chitosan beads. ABC: Alginate-bicarbonate-chitosan beads. The green line for the phage titres associated with the AB beads is obscured by the red line representing the titres associated with the A beads. Similarly, the orange line for the ABC beads is obscured by the purple line of the AC beads.

To confirm that each variety of beads was capable of releasing viable phages into SIF (which would be the desired effect should these beads be used therapeutically for Johne’s disease or Crohn’s disease), the number of phages present in bead–SIF mixtures at 37 °C was monitored for 6 h. It was observed that all four bead types released their maximum phage load within 90 min (Figure 6). Subsequently, the titres of the released phages remained constant, suggesting that they are not negatively impacted by the composition of the SIF. This is supported by the titre of the free phage control remaining consistent for the duration of the assay.

**Figure 7:**
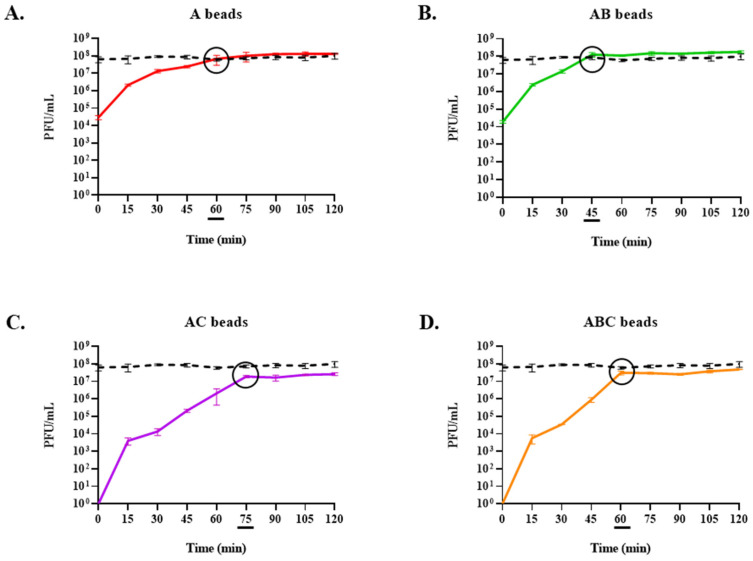
Phage release in simulated intestinal fluid (SIF) over 2 h. The moment the maximum phage titre is reached is indicated by a black circle on the graph and an underscore beneath the associated timepoint. Some error bars are obscured by the thickness of the lines. A control consisting of free phages that were used during the encapsulation process is indicated by a black dashed line in each graph. (**A**) Alginate (A) beads. (**B**) Alginate-bicarbonate (AB) beads. (**C**) Alginate-chitosan (AC) beads. (**D**) Alginate-bicarbonate-chitosan (ABC) beads.

To pinpoint the moment of maximum phage release and determine if this moment varied between bead types, this assay was repeated over 2 h. Monitoring the phage titres every 15 min revealed that the AB beads released their maximum phage load most rapidly (Figure 7B), with the maximum titre reached within 45 min. This was followed by the A (Figure 7A) and ABC (Figure 7D) beads at 60 min, and finally the AC beads at 75 min (Figure 7C). Notably, while no phages were detected at the beginning of the assay for the AC and ABC beads, the initial phage titres at the moment the A and AB beads were added were ~5 × 10^4^ PFU/mL, suggesting that their dissociation begins instantly (Figure 7A,B). At this point, AB stands out as a particularly desirable encapsulation configuration, as the total quantity of phages is released 25–50% sooner than the other formulations. However, as these results show that all beads could release viable phages, it was decided to see how each variety coped in SGF before making a final judgment on which bead is the most functionally appropriate.

### 3.6. Exposure of Encapsulated LOCARD to SGF and Release of Phages from Gastrically Treated Beads (GAB) in SIF

**Figure 8:**
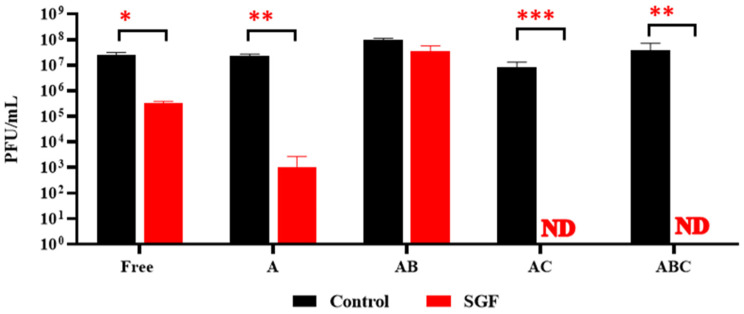
Recovery of phages following 90 min exposure to simulated gastric fluid (SGF). “Free” refers to a control consisting of free phages that were used during the encapsulation process. ND: not detected. A: Alginate beads. AB: Alginate-bicarbonate beads. AC: Alginate-chitosan beads. ABC: Alginate-bicarbonate-chitosan beads. Significance is illustrated on the graphs by asterisks as follows: no asterisk = *p* ≥ 0.05; * = *p* ≤ 0.05; ** = *p* ≤ 0.01; *** = *p* ≤ 0.001 The asterisk colour relates the significance value to the corresponding dataset.

**Figure 9:**
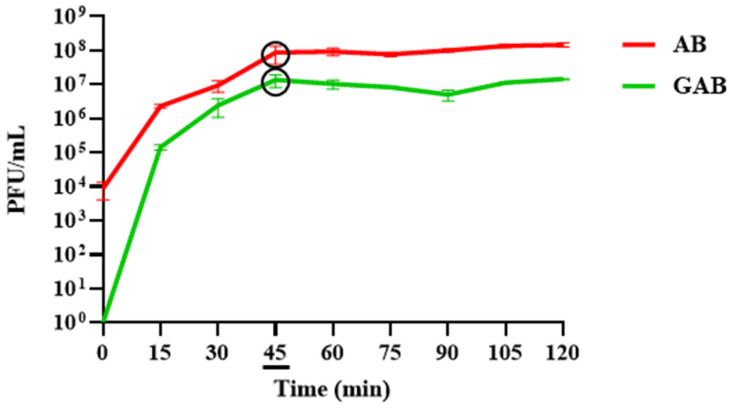
Release of viable phages from gastrically treated alginate-bicarbonate (GAB) beads in comparison to alginate-bicarbonate beads (AB) in simulated intestinal fluid (SIF). The moment the maximum phage titre is reached is indicated by a black circle on the graph and an underscore beneath the associated timepoint. Some error bars are obscured by the thickness of the lines.

To determine the impact of low pH and proteolytic action within a simulated gastric environment on the beads, the quantity of phages retained in each type was established following exposure to SGF for 90 min (alongside a control of free phages; Figure 8). This assay revealed that the AB beads were the only variety not significantly affected by the SGF (i.e., *p* ≥ 0.05). Meanwhile, the free phages were reduced by 1.88 log, the phages retained in the A beads were reduced by 4.36 log, and no phages were recovered from the AC or ABC beads (Figure 8). Based on this observation, it was decided to limit further investigations to include only the AB beads, as this clearly was the most robust formulation. The AB beads pre-exposed to SGF (GAB) were also capable of releasing viable phages into SIF, with the maximum phage load released at 45 min, in parallel with the untreated AB beads (Figure 9). The reduced viral load of the GAB is presumed to be an effect of the SGF pretreatment.

### 3.7. Reduction of M. smegmatis in SIF by Free and Encapsulated LOCARD

**Figure 10:**
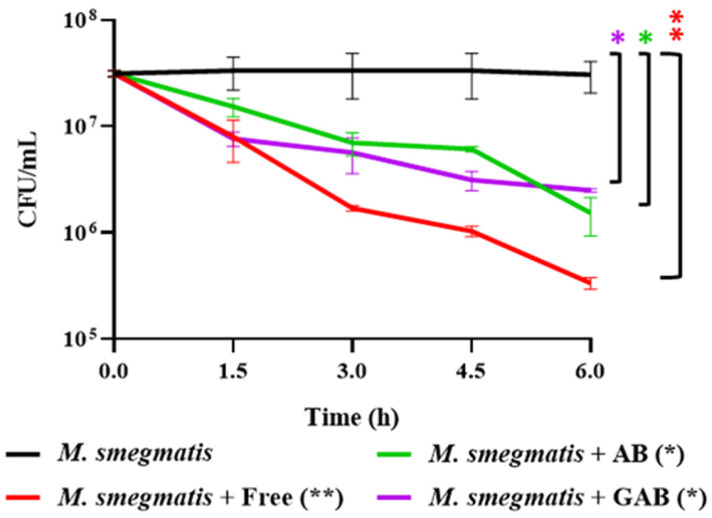
Reduction of mycobacterial populations in spiked simulated intestinal fluid (SIF) by free phages, alginate-bicarbonate (AB), and gastrically treated alginate-bicarbonate (GAB) beads. “Free” refers to a control consisting of free phages that were used during the encapsulation process. Significance is illustrated on the graphs by asterisks as follows: no asterisk = *p* ≥ 0.05; * = *p* ≤ 0.05; ** = *p* ≤ 0.01; The asterisk colour relates the significance value to the corresponding dataset.

The functionality of the AB beads was further explored by determining whether the phages released from AB and GAB beads could reduce the mycobacterial population in *M. smegmatis*-spiked SIF. Free phages, AB, and GAB beads were added to the spiked SIF, and mycobacterial numbers were monitored over 6 h (Figure 10). Unsurprisingly, the free phages were the most effective and significantly reduced the population of M. smegmatis by ~2-log (99%). Although less effective than the free phages, the phages released from the AB and GAB beads also significantly reduced mycobacterial numbers by ~1.5-log (96%) and ~1 log (90%), respectively.

### 3.8. Encapsulation of a Phage Cocktail as a Preliminary Demonstration of Multivalent Phage Beads

**Figure 11:**
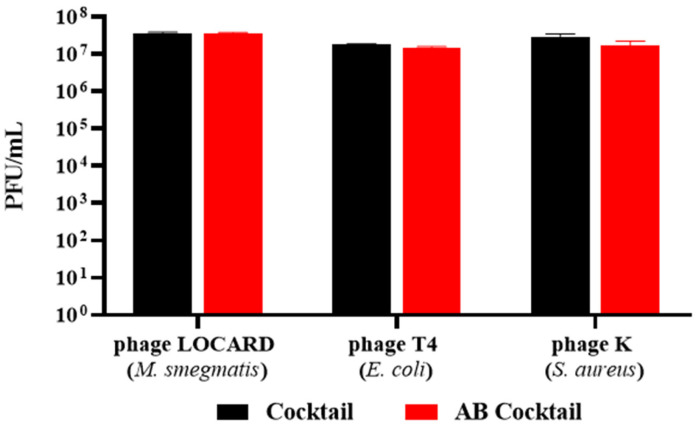
Recovery of phages from a three-phage cocktail (Cocktail) encapsulated in alginate-bicarbonate beads (AB Cocktail). No significant differences were noted during statistical analyses.

To examine the potential versatility of this approach, the encapsulation process was repeated with a three-phage cocktail (Cocktail) composed of phages with extremely distinct hosts. The phages LOCARD, T4, and K (targeting *M. smegmatis*, *E. coli*, and *S. aureus*, respectively) were co-encapsulated in the AB beads (AB Cocktail). There was no significant loss of particles for any of the phages during the encapsulation process (Figure 11), as similar titres of each phage were recovered from the beads compared to the titres present in the cocktail.

## 4. Discussion

Introducing phage-based methods of infection control to the agriculture sector could aid the reduction of antibiotic use in livestock [3]. In particular, prophylactic phage treatment of at-risk animals could confer a protective effect against difficult-to-treat infections, such as those associated with mycobacterial disease, as evidenced by Carrigy and colleagues [20]. Carrigy et al. successfully demonstrated phage-mediated prophylaxis by treating mice with a nebulised MP prior to aerosol challenge with *M. tuberculosis*. While these results are relevant for pulmonary mycobacterial illnesses, intestinal infections, such as Johne’s disease, are not likely to be impacted by the nasal delivery of MPs. Similarly, as evidence supporting the hypothesis that MAP is a triggering agent for Crohn’s disease continues to mount (e.g., 98% of blood samples from Crohn’s patients from Porto, Portugal, tested MAP-positive, and a subsequent study found that 30.8% of water sources in Porto contained MAP) [8,21], MPT could be a useful element of Crohn’s disease management strategies. Therefore, as Crohn’s is also an intestinal disease, creating a therapy suitable for oral administration would be favourable.

If oral delivery is to be considered, phages must encounter the gastrointestinal environment, and safeguarding the phage particles as they transit the stomach becomes paramount. One such method of safeguarding is alginate encapsulation. Alginate gels induced by the presence of Ca^2+^ form alginic acid at the interface of the alginate and low pH solutions. As pH values reach 5.5 and above, the alginic acid dissolves and the alginate disassociates [10,16]. Previous studies have exploited this pH-dependent solubility of pure alginate, and modified alginate gels like those formulated in this study to demonstrate the successful survival of phages targeting pathogenic bacteria in simulated gastrointestinal environments [13,14,15]. For this reason, this study focused on the alginate encapsulation of LOCARD, and is the first study to describe such an encapsulation approach for MPs.

Initially, a homogenous alginate–phage matrix was used as a “base” to create four varieties of beads (Figure 1 and Figure 2), which differed slightly in physical appearance (Figure 3). Each variety retained a large quantity of phages, although notably the chitosan treatment seemed to significantly reduce phage retention (Figure 4). This may be related to the chitosan solution itself, as the protein is only soluble in weak acids, and the 1% acetic acid used could be damaging the phages. The AC and ABC beads also suffered significant reductions in phage titre between Day 0 and Day 14 during a 4-week long storage assessment (Figure 5). Nonetheless, freshly produced AC and ABC beads retained a large quantity of phages. As chitosan may have proven beneficial when other factors were investigated, all four encapsulation configurations were added to SIF to determine if viable LOCARD particles would be released over time.

Every variety could release viable phages when exposed to SIF, and the phage titres were stable over 6 h (Figure 6). Notably, the initial titres of the SIF when the A and AB beads were added were ~4.5 log greater than the titres for the chitosan beads, suggesting that they begin to disassociate immediately. The full phage load of each variety of beads was released quite rapidly (within 90 min). The inclusion of bicarbonate appeared to accelerate the release, as the AB beads released their maximum viral load after 45 min in SIF (Figure 7B), while the A and AC beads did not achieve this until 60 and 75 min, respectively (Figure 7A; Figure 7C). This is indicative of a 25% more rapid release from the AB configuration. Likewise, the ABC beads released their phages 25% faster than the AC beads, suggesting that the inclusion of bicarbonate improves the dissociation of the alginate gels in SIF (Figure 7D). This observation is consistent with the understanding that an “antacid”, such as sodium bicarbonate, improves the dissolution and “buoyant raft” formation of alginate-based tablets used to alleviate acid reflux symptoms [18].

Ultimately, all beads were exposed to simulated gastric conditions—i.e., low pH and pepsin activity—for 90 min to determine if they could protect the LOCARD particles from the adverse environment. Intriguingly, the A beads lost a larger quantity of phages than the free phage control, but they still retained more phages than the AC or ABC varieties, from which no phages were recovered (Figure 8). The latter is consistent with other studies that found that alginate-chitosan beads are insufficient for >1 h exposure to SGF, as summarised by Briot et al. [22]. Only the AB beads were not significantly impacted by the SGF, which is also likely related to the fact that alginate–bicarbonate matrices are frequently used to soothe acid reflux [18,23]. Similar observations were noted when a more complex SGF was trialled based on the INFOGEST system [24]. As can be seen in Figure 9, GAB beads release viable phage particles as readily as the untreated AB beads. At this point, it seemed clear that the AB beads were the most efficient and robust formulation of encapsulated LOCARD.

Attempts were made to apply elements of mechanical digestion to the AB-SGF suspensions using a Stomacher 400CIRCULATOR (Seward, Worthing, West Sussex, UK) to further challenge this configuration of encapsulated LOCARD. However, no phages were recovered from the stomached samples. It is important to note that Stomachers are designed for roughly homogenising (typically food) samples in order to permit microbiological profiling [25]. Therefore, they are not an appropriate apparatus to generate the desired peristaltic force. The model described by Li et al. [26] would be far more suitable.

In the absence of this model, more GAB beads were generated using the original method described in the Section 2, and added to SIF spiked with *M. smegmatis*. The rapid growth of this surrogate organism facilitated the generation of Figure 10 and Figure 11, which would have been difficult with MAP, given its minimum 16-week culture period [5]. These experiments were conducted with the understanding that the results would be translatable to a phage with activity against MAP. The phages released from the GAB beads significantly reduced mycobacterial numbers by ~90%. While the free phages were the most effective at reducing the *M. smegmatis* population, the differences in efficacy are most likely related to the free phages having a “head start” based on two main factors: (1) the total phage load of the encapsulated phages was not released until approximately 45 min after the initial timepoint, based on the data from Figure 9; and (2) the gastric treatment of the GAB likely reduced the viable phage numbers by 0.5–1 log. Ideally, this phase of digestion would also be examined in a more sophisticated intestinal model, such as the previously mentioned Li et al. [27] model, or the distal colon model described by Lawrence et al. [28], but in its absence, these preliminary results suffice as a proof-of-concept demonstration.

This work provides clear evidence that encapsulated phages can survive low pH and pepsin activity, and viable phages can be released into SIF and ultimately reduce mycobacterial populations. Overall, these results suggest that the suppository use of encapsulated phages may be an option, as all beads release phages into SIF. A recent study has demonstrated the protective and preventative efficacy of suppositories composed of *E. coli* phages and probiotics for eliminating *E. coli*-related diarrhoeal disease in calves within 48 h [29]. This suggests that MP suppositories may help reduce and/or prevent the diarrhoeal symptoms associated with Johne’s disease. When considering human patients experiencing MAP-triggered Crohn’s disease, however, oral delivery may be preferable due to personal comfort [30]. Ideally, future work would involve small-scale in vivo studies to optimise the therapeutic and/or prophylactic application of the AB beads, particularly in young ruminants. Similarly, multivalent phage cocktails featuring phages that target multiple pathogens could be prepared and administered to investigate the control of several relevant infections at once, as suggested by the successful encapsulation of a diverse cocktail in this study.

## Data Availability

Data are contained within the article.
